# Causal inference methods for systematic reviews

**DOI:** 10.5195/jmla.2026.2253

**Published:** 2026-10-01

**Authors:** Madelyn Glymour

**Affiliations:** 1 glymourm@duq.edu, Medical Librarian, Gumberg Library, Duquesne University, Pittsburgh, PA

**Keywords:** Systematic review, causal inference, methodology, confounder bias, collider bias

## Abstract

Systematic reviews are frequently used to ask and answer causal questions, and decisions made throughout the review process can introduce bias because of the causal relationships between variables of interest, even when the question being asked is not itself causal. However, current systematic review best practices do not consider most of these impacts.

The author presents an introduction to the basic principles and terminology of causal inference as apply to systematic reviews and discusses how some simple causal inference concepts can be used to create clearer and less biased research questions, search strategies, eligibility criteria, and data analysis. By taking into account confounder and collider bias, and by paying careful attention to the differences in assumptions made in intervention- and non-intervention-based reviews, health science librarians can aid systematic review teams in minimizing the risk of bias, and in appropriately analyzing and assessing that risk.

## INTRODUCTION

Systematic reviews, like many evidence-based practice (EBP) tools, are often used to ask and answer causal questions. The PICO framework, frequently used and recommended to guide question formation in reviews, is focused on the outcomes of interventions. Researchers want to learn the effect of interventions on the patient, to better inform the practices of clinicians.

But the causal element of systematic reviews is not limited to question formation. Just as in a primary study, nearly every step in the systematic review process can have a profound impact on the reliability of the information procured, even when the question being asked is itself not causal. Decisions made about exclusion criteria, search strategy, and data analysis can introduce not just selection bias, but other subtler inaccuracies, because of the causal relationships between the factors being measured and studied. In particular, any review of non-intervention studies, such as cohort and case-control studies, is at risk for introducing bias both through the individual studies collected and through the process used to collect them. However, many current EBP best practices for systematic review do not take into account most of those impacts [1].

Librarians are often included on systematic review teams in order to help write and run the search strategy. However, that is not the only role that librarians can or do take in systematic reviews; librarians are often called on to advise or assist with question formation, protocol development, database selection, and other elements of a review that can be profoundly impacted by causal considerations [2]. Outside of reviews proper, health sciences librarians often have a significant role in teaching researchers and clinicians the EBP tools that form part of the systematic review process, including research question formation, critical appraisal, and some basic statistics and outcomes reporting needed for evidence evaluation [3]. As both practitioners and teachers of systematic review methods, it is therefore vital that health sciences librarians understand the ways that causal relationships affect the input and output of these tools.

*Causal inference* is an approach to statistics and data analysis that is concerned with determining whether associations within data are due to causal relationships. This paper presents some basic causal inference principles that are applicable to the systematic review process and discusses how health sciences librarians can use them to create and teach clearer and less biased research questions, search strategies, eligibility criteria, and data analysis.

## DIRECTED ACYCLIC GRAPHS

One of the foundational tools in the causal inference toolbox is the *causal graph*. A causal graph represents the causal relationship between variables; each variable is a node in the graph, and arrows (*directed edges*) between the nodes represent the direction of causality [6]. A *directed acyclic graph* (DAG) is one in which none of the arrows form a cycle.

Causal graphs are best understood as a visual depiction of the *data-generating process*: the story of how the variables in question came to take on the values we see in the data. Because of that, a causal graph implies consistent and testable independence relationships between variables in the data. Two variables are dependent in a data set if values of one variable are associated with, or predictable from, changes in the other. Two variables are independent if their values are not related. Based on a causal graph, we can predict whether two variables will be dependent or independent of each other. We can also predict whether they will be independent *conditional on* a third variable, that is, after controlling for or stratifying on values of another variable in the graph. These relationships are called *conditional independence relationships*.

Conversely, if we know the causal graph underlying a data set, we can use the graph to rigorously make decisions about how to interpret the data. Or, in the case of systematic reviews, how to seek out data while minimizing the risk of certain kinds of bias. This paper will discuss the inferences we can make based on two different kinds of graph structures: *confounders* and *colliders*.

**Figure 1:**
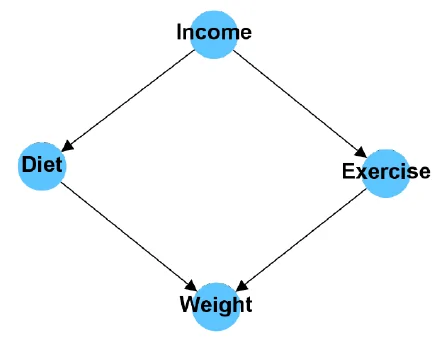
A directed acyclic graph (DAG). In this graph, Income is a confounder of Diet and Exercise, and Weight is a collider of Diet and Exercise. Income is a parent of Diet and Exercise, and an ancestor of Weight. Weight is a child of Diet and Exercise, and a descendant of Income.

Confounders occur when one variable is the *parent* (direct cause) of two other variables; the parent variable *confounds* the relationship between the children [5]. The child variables are dependent in general, but are independent conditional on the confounder. For instance, income level has a significant impact on both diet and exercise [7, 8]. It therefore confounds the relationship between those two variables. People with sedentary lifestyles might be more likely to eat less healthy food, not because either diet or exercise is cause of the other, but because both are effects of lower income. *Confounder bias* occurs when researchers fail to control for a confounder, causing two unrelated variables to appear related.

Colliders occur when one variable is the *child* (direct effect) of two other variables [4]. When that happens, arrows from the two parent variables “collide” at the child variable. For example, diet and exercise are both factors that influence body weight. In a causal graph, weight is a child of both variables, and thus a collider. Colliders occur often in health care, and can create serious bias, but they are less widely understood than many other sources of bias [9].

## QUESTION FORMATION

As part of the process of developing a search strategy and advising researchers on methodological concerns, librarians are frequently called upon to participate in and comment on the development of the research question itself [2]. This often takes the form of helping to define the scope of the review: what populations, subpopulations, specific treatments, article types, and so on will be included. Decisions made in shaping the research question have major impacts on the outcome of the review, including the amount of bias—because the research question is one significant place where confounder bias might make its way into a review.

The PICO framework (patient/problem/population, intervention, comparison/control, outcome) is the most widely used system for constructing a research question for systematic reviews, recommended in authoritative resources such as The Cochrane Handbook [10]. Although the PICO framework appears simple, constructing a question using it can be deceptively complex. The elements of a PICO question, particularly population, can be multifaceted. When constructing a research question about the relationship between brain rest (intervention) and recovery time (outcome) from concussion (population), for instance, should our population be all people with concussion? Should it be people with minor concussions or major concussions only? Should we limit our population to *athletes* with concussion, or children with concussion, or people with pre-existing migraine and concussion? In causal inference terms, when we’re determining whether “brain rest” and “concussion recovery time” are dependent, should we condition on concussion severity, age, or comorbidity?

Logic models, or theories of change, are often cited as a tool for making these “lumping” vs. “splitting” decisions in literature reviews [10, 11]. Logic models are similar to a causal graph in that they provide a visual organization of the flow of causation. But there is little practical advice on how to construct or interpret a logic model causally, or how to use one to identify confounders. And yet, because of confounders, lumping and splitting decisions can have a profound impact on the results that a systematic review produces.

Consider, for example, that you are consulting on a systematic review of the effectiveness of a new kidney stone treatment. Together, you construct a question using the PICO framework: “Does New Treatment have a higher single-treatment clearance rate than Old Treatment for kidney stones larger than 2 cm?” The researchers’ justification for limiting the population is that stone size can have an impact on treatment success, and therefore it is sensible to examine the new treatment’s impact on the most severe disease. But after an initial pilot search returns a relatively small number of results, the researchers want to consider expanding the population to stones of all sizes: “Does New Treatment have a higher single-treatment clearance rate than Old Treatment for kidney stones?” How would you advise them? What tools would you use to do so?

Causal inference provides a rigorous method for deciding lumping and splitting questions. This method, called the *backdoor criterion*, is used to decide whether or not we should condition on a variable when determining the relationship between a treatment and an outcome (12). The backdoor criterion tells us that we should only condition on that variable if it lies on a “backdoor” path between the two—that is, if in the DAG representing the causal relationships, the variable lies on a path between treatment and outcome where the arrows point *towards* the treatment and *out of* the outcome.

The first step in using the backdoor criterion to decide on the kidney stone problem would be to advise the researchers to construct a formal causal graph, using their own subject matter expertise and a background review of the subject. In doing so, they would hopefully come across a 1986 paper by Charig et al. that performed an observational comparison of three different types of kidney stone treatments [13], whose results are shown in Table 1.

**Table 1:** Success rate of open surgery vs. PCNL in removing kidney stones (13).

	Open Surgery success rate	PCNL success rate
Stone diameter < 2cm	81/87 (93.1%)	235/270 (86.7%)
Stone diameter ≥ 2cm	192/263 (73.0%)	55/80 (68.8%)
**Total (all stones)**	**273/350 (78.0%)**	**289/350 (82.6%)**

According to their data, open surgery was more effective at clearing kidney stones than percutaneous nephrolithotomy (PCNL) in patients with small stones (93.1% of stones were cleared with open surgery, vs, 86.7% of stones cleared with PCNL). And open surgery was more effective at clearing kidney stones than PCNL in patients with large stones (73% of stones cleared with open surgery, vs. 68.8% of stones cleared with PCNL). Conditional on stone size, open surgery was more effective than PCNL. But when the data of small stone and large stone patients were aggregated, the results were reversed: PCNL (82.6%) cleared stones more frequently than open surgery (78%). In this case, the question of whether to lump or split the population is critical to the outcome.

Charig et al.’s results are both illustrative of the importance of thinking critically about lumping vs. splitting questions and a helpful indicator of how the researchers’ causal graph should be constructed. It was eventually determined that the seemingly paradoxical results that Charig et al. found were because of a particular kind of confounder in the DAG, shown in Figure 2 [14].

**Figure 2:**
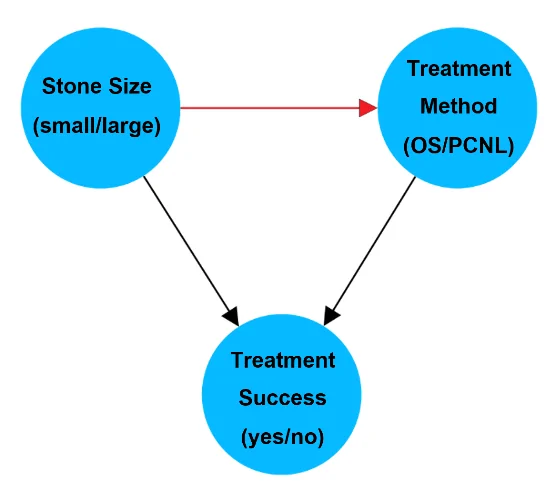
A DAG representing the causal relationships between the variables in Charig et al.’s study. Stone Size is a confounder of Treatment Method and Treatment Success. The “backdoor” arrow into Treatment Method is highlighted in red.

The confounding occurred because the stone size and treatment method were both a cause of treatment success, *and* stone size was a cause of treatment method. Doctors were more likely to prescribe the less aggressive, newer treatment to smaller, easier-to-treat stones, which biased PCNL in favor of a high stone clearance rate. Once we see the causal graph, we can see that Stone Size satisfies the backdoor criterion, because it is not caused by Treatment Method, and it blocks the only “backdoor” pathway (one in which there is an arrow into Treatment Method) between Treatment Method and Treatment Success. In order to get the correct estimate of the efficacy of the treatment, we therefore need to condition on stone size—separate our analysis, our results, and crucially, our research question, into small-stone and large-stone categories. So for our hypothetical researchers, we would advise them to either use their original research question, or if they feel they need to broaden the scope, to include another limitation that addresses the problem of confounding, such as only considering randomized controlled trials.

In addition to confounder bias, the backdoor criterion can also help to avoid *overadjustment bias* in question formation. Overadjustment bias is the opposite of confounder bias; it occurs when controlling for a variable creates bias in data analysis. Not only is it not feasible to condition on every possible confounder in our population, controlling for the wrong variables can introduce bias in a number of ways. The most immediately obvious is that if a presumed confounder is instead a *mediator*—a variable that is part of the process by which a treatment affects an outcome—then by controlling for it, we can make the impact of the treatment on the outcome look smaller than it really is.

For instance, regular exercise reduces heart attack risk. It does so in part by reducing cholesterol, which in turn reduces heart attack risk (15, 16). In a study of the effect of an exercise program on heart attack, controlling for cholesterol would reduce the effect size. Sometimes that’s desirable; *mediation analysis* is a type of data analysis in which researchers use similar techniques to determine the amount that particular mediators contribute to overall effect. But if we treat cholesterol as a confounder, we will introduce bias that makes exercise look less effective than it truly is at reducing heart attack risk (See Figure 3). If we were consulting with researchers performing a review of the overall impact of exercise on heart attack risk, we could use the backdoor criterion to advise them not to include cholesterol as an element of the population in their research question.

**Figure 3:**
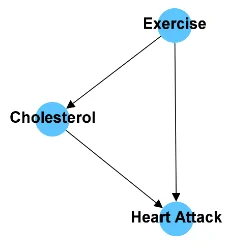
A DAG of the causal relationships between exercise, cholesterol, and heart attack risk. Cholesterol mediates the effect of exercise on heart attack. It does not lie on a path with a “backdoor” into Exercise.

## COLLIDER BIAS

Another common form of overadjustment bias, and one which is frequently overlooked, is *collider bias*. When we condition on a collider, or on a child of a collider, we create an association between the two parent variables, even if none existed before. The parents are independent in general, but dependent conditional on the collider.

Collider bias often appears as a form of selection bias. For instance, collider bias appeared in early COVID-19 studies [17]. Early studies were performed using the available testing data, which at the time featured a significant oversampling of severe cases (who were more likely to be hospitalized and therefore tested) and healthcare workers (who were more likely to test frequently, due to their risk) [18].

**Figure 4:**
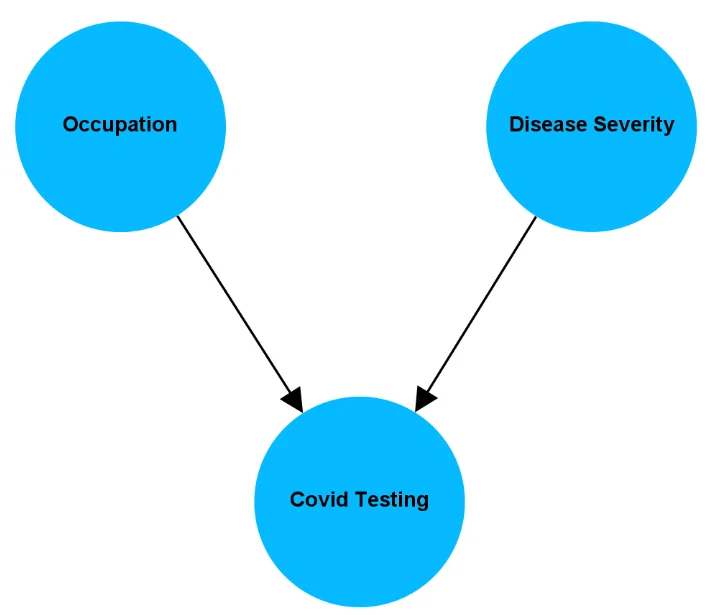
A DAG representing the causal relationships between occupation, COVID severity, and COVID testing early in the COVID-19 pandemic.

The use of those studies created collider bias, in which it appeared that being a healthcare worker was protective against severe covid, because in that sample, healthcare workers were significantly less likely to have severe covid than non-healthcare workers.

Any situation in which we limit or stratify our sample has the potential to introduce collider bias, if decisions are not made in a careful and informed way. In PICO-based systematic reviews of experimental studies, collider bias is of most concern during data analysis, where teams that stratify their results based on study quality [19] or symptom or disease severity [20] can create a non-existent association between the variables they’re interested in.

However, many systematic reviews include studies with non-experimental designs, like cohort and case-control studies. And not all systematic reviews are interested in the outcome of an intervention. Some are interested in the effects of an exposure (for instance, lead exposure, or diabetes) on an outcome (for instance developmental delays, or heart attack). Others are interested in the accuracy of a diagnostic test for a particular condition. These non-intervention reviews are at risk of collider bias at nearly every stage of the review.

### Question Formation

In question formation, collider bias may arise if the population is causally linked to the outcome and the exposure. For instance, a review with the research question, “Does gallbladder inflammation exacerbate type II diabetes among hospitalized patients?” might induce collider bias by limiting the population to inpatients, because both the exposure (inflamed gallbladder) and the outcome (diabetes) are causes of being hospitalized [20]. This might make it appear that gallbladder inflammation is associated with less severe diabetes—because the sample would include a large number of people with severe diabetes but no gallbladder problems (who were hospitalized because of diabetes), a large number of people with mild diabetes and inflamed gallbladders (who were hospitalized because of gallbladder disease) and a very small number of people who just happened to have both gallbladder inflammation and severe diabetes. A better framing of this question might be setting-agnostic; that would capture the population of patients with mild diabetes and no gallbladder inflammation, who are unlikely to be hospitalized. This is particularly important to keep in mind when using alternative question frameworks such as SPICE (setting/population/intervention/comparison/evaluation) or PCC (population/concept/context) that more explicitly take setting into account.

### Search Strategy

Collider bias may occur in the search strategy, if any database limits or search filters used are causally linked to elements of the research question. For instance, a team performing a review of the effect of kidney disease on heart attack risk might decide that they want to limit their search to clinically useful journals. But clinically useful journals are more likely to study and report on subjects in a clinical context, and health-care utilization is a collider [9]. Both kidney disease and heart attack make people more likely to interact with physicians and other clinicians, so if search results are biased in favor of health care settings, we might again find a spurious association in which it looks like kidney disease is associated with a lower risk of heart attack, because we are missing out on the population of patients with low heart attack risk and no kidney disease.

### Study Screening

In study screening, eligibility criteria might create a collider. For instance, study setting is a common eligibility criterion in systematic reviews, but excluding studies performed outside (or inside) a hospital can introduce collider bias. Before creating an exclusion criterion, review teams should assess whether it might be a downstream effect of the exposure, the outcome, or both.

### Data Analysis

Most vitally, collider bias may occur in the data of the synthesized studies. Collider bias is a risk of any non-experimental study, even when the study draws from a very large sample, especially if that sample self-selects in any way. For instance, studies of the link between genetic variation and phenotypes (such as the expression of a genetic disease, or disease severity) often rely on gene banks for their data. But genes may influence factors that make people more or less likely to participate in a gene bank, making study participation itself a collider [21]. Most risk-of-bias assessment tools, however, do not ask about the possibility of collider bias in the study being assessed [1].

Collider bias is particularly troubling because it can create the appearance of associations that do not actually exist. Approaching a non-intervention systematic review with an understanding of causal inference can help reviewers to construct reviews that minimize the risk of creating those spurious correlations, and to appropriately analyze and report that risk. Although librarians are not always asked to collaborate on all of these stages of a review, when they are, they can help the teams they work with to minimize collider bias by encouraging the construction of a well-founded causal model before decisions are made about databases, exclusion criteria, and search filters. Librarians advising on risk-of-bias tools for non-intervention reviews can minimize collider bias by suggesting tools, such as ROBINS-I [22], that ask about the possibility of overadjustment.

## DISCUSSION

Systematic reviewing, and evidence-based practice writ large, are a discrete set of practical tools for performing research and making clinical decisions. But they could equally be said to be a mindset: a way of viewing data, research, and literature search that aims to understand the source of information, appraise its reliability, and minimize bias in its analysis and reporting.

Individual health sciences librarians can use causal inference to improve the evidence synthesis reviews that they participate in, and their instruction on the component EBP tools of reviews, by incorporating causal inference into that mindset:

- Encourage the development of a well-founded causal model before making decisions about the structure of a review, particularly in non-intervention reviews.
- When discussing question formation, in addition to considering the feasibility, relevance, and logic of the question, make sure that team members or students understand the potential causal implications of limiting or not limiting the population. In non-intervention reviews, consider whether the population might be a common effect of the exposure and outcome.
- When participating in non-intervention or non-experimental reviews, consider collider bias when deciding on and reporting on search strategies and eligibility criteria. During search, consider whether any filters or limits used might be a downstream effect of a collider such as healthcare utilization. During screening, pay attention to exclusion criteria that may be causally connected to the exposure and outcome. During data analysis, recommend risk of bias tools that ask about overadjustment and collider bias.

The specific EBP guidelines and tools used and recommended for systematic reviews could also benefit from incorporating causal inference methods. Critical appraisal and risk-of-bias tools of non-intervention studies might include questions appraising collider bias. Question formation frameworks might benefit from structured guidance on how to select elements in causally consistent ways. Better guidance could be developed for when logic models are and are not appropriate tools for assessing causation and justifying a theory of change, and for how to construct them in ways that clearly lay out their causal implications.

When a librarian’s role in a systematic review grows outside the boundaries of designing the search, the role often becomes that of the custodian and record-keeper of the review’s methodology—to understand and advise on how the decisions made in the design, implementation, and reporting of the review may impact its results, and to ensure that all of those decisions are documented so that others in the future can understand what happened. Librarians in these roles become keepers of the *how* and the *why*: How did this review come to be? Why did we use these keywords? How did we find these studies? Why did we decide to keep some and discard others?

*How* and *why* are causal questions, and the best-prepared librarian will be the one who knows not just the answers, but what the answers mean. With the tools causal inference provides, we can prepare ourselves to give knowledgeable, evidence-based guidance to the researchers we work with, to help them get and understand the answers they’re looking for.

## Data Availability

There are no data associated with this article.
